# Can artificial tears prevent *Acanthamoeba* keratitis? An in vitro approach

**DOI:** 10.1186/s13071-018-2639-5

**Published:** 2018-01-22

**Authors:** Angela Magnet, Thiago Santos Gomes, Carmen Pardinas, Natalia Garcia de Blas, Cruz Sadaba, Eugenia Carrillo, Fernando Izquierdo, José Manuel Benítez del Castillo, Carolina Hurtado, Carmen del Aguila, Soledad Fenoy

**Affiliations:** 1 Facultad de Farmacia, Universidad San Pablo CEU, CEU Universities, Madrid, Spain; 2 Facultad de Medicina, Universidad San Pablo CEU, CEU Universities, Madrid, Spain; 3 Servicio de Oftalmología, Hospital Clínico San Carlos, Universidad Complutense, Madrid, Spain; 4 Instituto de Salud Carlos III, Centro Nacional de Microbiología, WHO Collaborating Centre for Leishmaniasis, Madrid, Spain; 5 CAPES Foundation, Ministry of Education of Brazil, Brasília, DF 70040–020 Brazil

**Keywords:** *Acanthamoeba*, Prophylaxis, Artificial tears, Preservatives, Amoebicidal effect

## Abstract

**Background:**

The use of contact lenses has increased in recent years as has the incidence of Dry Eye Syndrome, partly due to their use. Artificial tears are the most common treatment option. Since these changes can facilitate *Acanthamoeba* infection, the present study has been designed to evaluate the effect of three artificial tears treatments in the viability of *Acanthamoeba* genotype T4 trophozoites. Optava Fusion™, Oculotect®, and Artelac® Splash were selected due to their formulation.

**Methods:**

Viability was assessed using two staining methods, Trypan Blue stain and CTC stain at different time intervals (2, 4, 6, 8 and 24 h). Trypan Blue viability was obtained by manual count with light microscopy while the CTC stain was determined using flow cytometry.

**Results:**

Trypan Blue staining results demonstrated a decrease in viability for Optava Fusion™ and Artelac® Splash during the first 4 h of incubation. After, this effect seems to lose strength. In the case of Oculotect®, complete cell death was observed after 2 h. Using flow cytometry analysis, Optava Fusion™ and Oculotect® exhibited the same effect observed with Trypan Blue staining. However, Artelac® Splash revealed decreasing cell respiratory activity after four hours, with no damage to the cell membrane.

**Conclusions:**

The present study uses, for the first time, CTC stain analyzed by flow cytometry to establish *Acanthamoeba* viability demonstrating its usefulness and complementarity with the traditional stain, Trypan Blue. Artelac® Splash, with no preservatives, and Optava Fusion TM, with Purite®, have not shown any useful amoebicidal activity. On the contrary, promising results presented by Ocultect®, with BAK, open up a new possibility for *Acanthamoeba* keratitis prophylaxis and treatment although in vivo studies should be carried out.

## Background

*Acanthamoeba* spp. are one of the most commonly isolated amoebae in environmental samples.They have a cosmopolitan distribution and can act as both opportunistic and non-opportunistic pathogens [1]. These amoebae have been isolated from soil, dust, air, seawater, swimming pools, sewage, sediments, air-conditioning units, domestic tap water, bottled water, dental treatment units, hospitals, dialysis apparatus, eyewash stations, contact lenses and their cases and as contaminants in bacterial, yeast and mammalian cell cultures [2, 3]. *Acanthamoeba* species present two morphological stages in their life-cycle: a vegetative trophozoite stage, in which they are active and reproduce by binary fission, and a cyst stage that is resistant to environmentally adverse conditions [4, 5].

These free-living amoebae are typically harmless to humans, but in rare instances can cause severe infections. One of these infections, *Acanthamoeba* keratitis (AK), is an ulceration of the cornea which, if not treated promptly, can cause extensive ocular damage, leading to loss of vision acuity, blindness and possible enucleation [4, 6–8].

In recent decades, there has been a remarkable rise in the number of diagnosed AK cases, mostly due to an increase in the number of contact lens (CL) wearers [9, 10]. Most of these cases affect immunocompetent CL wearers and result from poor hygiene practices as well as the failure to comply with recommended cleaning and disinfection procedures, rinsing with tap water or homemade saline solutions, showering while wearing lenses and the extended use of disposable CL [11].

Contact lenses wear alone is frequently associated with symptoms of ocular irritation, including dryness, discomfort, soreness and tiredness [12]. Sometimes these signs of ocular surface impairment resemble dry eye conditions in non-lens wearers. Dry eye syndrome (DES) is a disorder of the tear film due to tear deficiency or excessive tear evaporation, which can cause damage to the interpalpebral ocular surface. It is also associated with symptoms of ocular discomfort, and contact lens dry eye is considered a sub-classification of this syndrome [13, 14].

The sole presence of a CL on the eye affects the nature of tear film dispersal. A reduction in the pre-lens tear film lipid layer and an increase in tear film evaporation are attributed to CL wear, resulting in the onset of dryness. Additionally, the disruption of the tear film by the CL may lead to compromised functional visual acuities, reduced wear time, and an increased risk of ocular surface desiccation, bacterial binding and infection [12]. These alterations in the corneal epithelium produced both by DES and CL wear, can create a possible entry point for ocular surface invasion [15]. For example, several in vivo studies indicate that corneal trauma is a prerequisite for AK, as animals with intact corneas (i.e. epithelial cells) do not develop this infection [5].

For the treatment of these symptoms, rewetting drops are traditionally the most common first-line option. However, technological advances have led to the development of artificial tear solutions, also known as lubricant eye drops, which mimic the tear film function and protect the ocular surface from dryness. Recently, several artificial tear solutions have been produced to reduce these symptoms. They account for at least $540 million in annual sales globally and are currently the mainstay of therapy of DES due to their noninvasive nature and reduced side effect profile [12, 16, 17].

Moreover, these artificial tears are formulated with different preservative compounds that may present useful properties in preventing ocular infections. In this study, the potential amoebicidal effects of artificial tear solutions were evaluated for the first time in order to establish their usefulness in the prevention of AK.

## Methods

### *Acanthamoeba* culture

*Acanthamoeba* spp. strain USP-CR5-A35 genotype T4, which was originaly isolated form a Spanish keratitits patient, was used to study the effect of the artificial tears analyzed [18]. Axenic cultures were grown in PYG medium (0.75% protease peptone, 0.75% yeast extract and 1.5% glucose with 40 μg gentamicin per milliliter) at 28 °C without shaking. After 24 h of culture, the culture flask was incubated on ice for 5 min to favor amoebae de-attachment and then trophozoites were collected with the help of a pipette, washed twice with NEFF saline [19] and the concentration was adjusted in NEFF to 4 × 10^5^ amoebae/ml.

### Tested artificial tears

Three different commercially available artificial tears were chosen for this study due to the different types of preservatives (detergent or oxidative) or the lack of them (Artelac® Splash, Bausch & Lomb, Berlin, Germany) in their formulation. Characteristics of the different tears are shown in Table 1. Benzalkonium chloride (BAK) which interrupts the lipid component of cell membranes acting as an antibiotic and amoebicidal) is a detergent found in Oculotect® (Laboratoires Alcon, Kaysersberg, France) [20]. Purite® (Allergan, Westport, Ireland) is a stabilized oxychloride complex with chlorine dioxide, chlorite and chlorate found in Optava Fusion™ (Allergan, Westport, Ireland). When excited by light, they produce water, oxygen, sodium, and chlorine free radicals. These radicals interact with the pathogen membrane causing death. To our knowledge, Purite® has never been tested for *Acanthamoeba*.

**Table 1:** Characteristics of the artificial tears studied and cytometry results are shown as viability percentage

	Components	Preservative	% viability (TB); % viability (CTC); % altered morphology (CTC)			
			2 h	4 h	6 h	8 h
Oculotect® 50 mg/ml (Novartis)	Povidone K25; sodium hidroxide	Benzalkonium chloride (BAK)	3.6; 4.9; 0	1.8; 7.3; 0	5.5; 1.5; 0	2.5; 4.1; 0
Optava Fusion™ (Allergan)	Sodium hyaluronate; sodium carboxymethylcellulose; glycerin; erythritol	Purite®	44.3; 44.5; 16.6	46.5; 26.7; 6.8	79.0; 34.2; 19.8	85.7; 31.0; 28.7
Artelac® Splash (Bausch+Lomb)	Sodium hyaluronate; sodium chloride; potassium chloride; sodium phosphates	None	52.0; 58.8; 3.3	58.0; 34.6; 12.6	72.0; 52.8; 4.1	81.5; 10.8; 10.9

### Effect of artificial tears against *Acanthamoeba*, culture and microscopic observation

In T25 culture bottles, 2.5 ml of the amoebae suspension (USP-CR5-A45 at 4 × 10^5^ amoebae/ml) was cultured with 2.5 ml of the artificial tears. Non-effect control was performed with NEFF saline. These cultures were incubated at 33 °C for 2 h, 4 h, 6 h and 8 h as previously described [20–22]. Before culture collection, the appearance of the *Acanthamoeba* trophozoites was checked under light microscopy at 400× (Nikon Eclipse TS100, Nikon Instruments Europe B.V.). Cultures were collected and washed with PBS by centrifugation at 1500× *rpm* for 10 min. After, amoebae were suspended in 1 ml of PBS and divided into two aliquots for the viability test. Culture with the artificial tears was performed in triplicate for the four different incubation times.

### Viability tests

#### Trypan blue stain

Trypan Blue 0.4% (BioWhittaker®, Walkersville, USA) was used 1:1 with the amoebae suspension to establish *Acanthamoeba* viability after incubation with the different tears. Trypan Blue is a vital stain that colors only dead cells. Living amoebae have a refringent appearance under light microscopy while dead amoebae exhibit a blue color. Cells were counted using a BRAND® counting chamber BLAUBRAND® Neubauer pattern (Sigma-Aldrich, Merck KGaA, Darmstadt, Germany).

#### Flow cytometry

To establish *Acanthamoeba* viability, Bacstain-CTC Rapid Staining Kit (Dojindo,Kumamoto, Japan) was used to stain viable amoebae. CTC (5-cyano-2,3-ditolyl-tetrazolium chloride) is a stain used to measure bacterial viability. CTC is a soluble reagent which, when it interacts with the respiratory system, gains a proton and becomes an insoluble product, CTC formazan. This product is fluorescent, a characteristic that allows it to be used in flow cytometry. Kobayashi et al. [23] first described the use of this product for *Acanthamoeba* viability in a fluorometric assay.

Amoebae were cultured with CTC for 30 min and then washed and fixed with PBS-1% formalin. Parasites were identified based on forward/side scatter values and a total of 10,000 events was obtained by FACScalibur cytometer using the CELLquest software. The fluorescence was evaluated in FL3 histograms. Cytometer files were analyzed using FlowJo software (Version 7.6.5, Tree Star, Ashland, USA).

## Results

### Microscopic observation

**Fig. 1:**
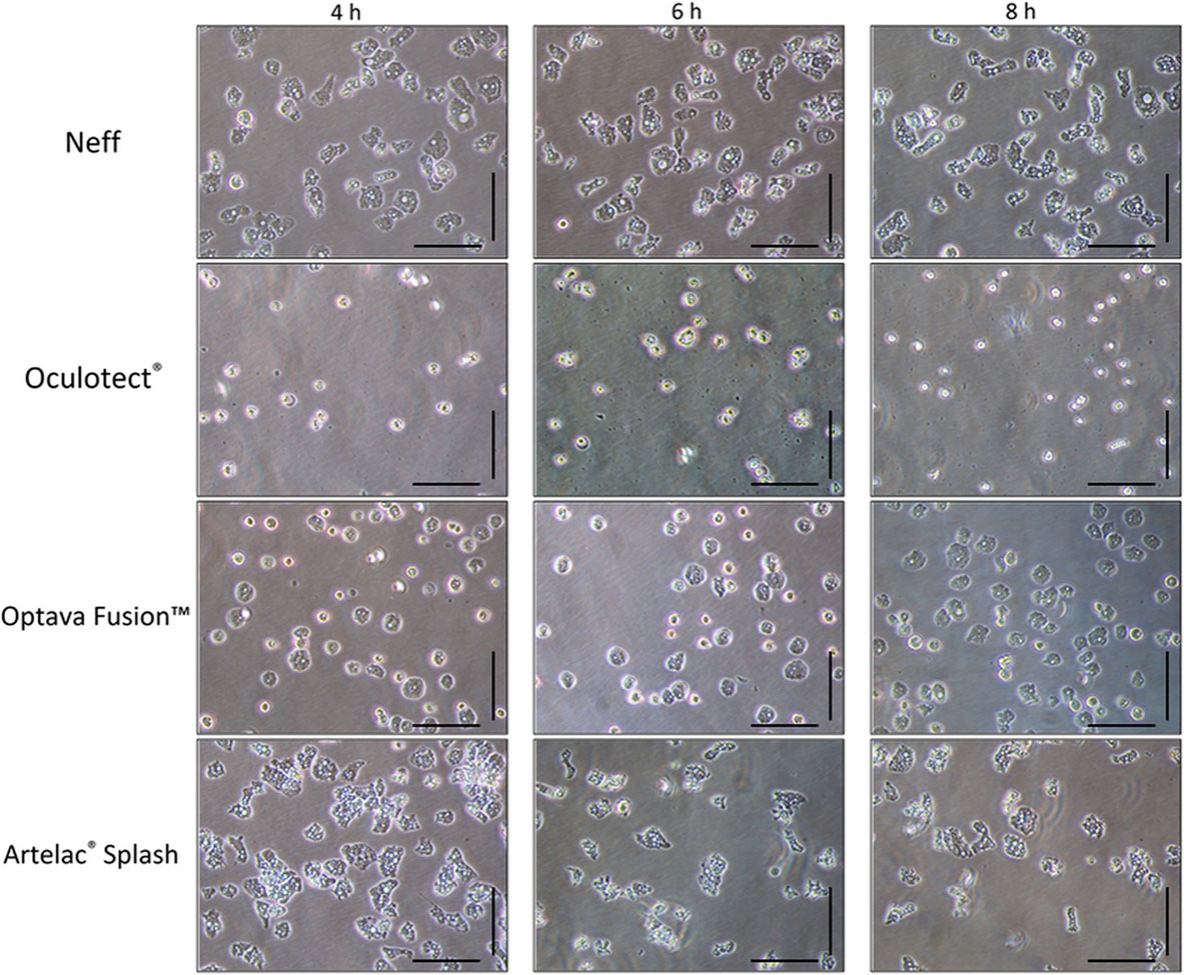
*Acanthamoeba* culture appearance at 4, 6 and 8 h of incubation with the studied artificial tears. *Scale-bars*: 100 μm

Cultures were observed using an inverted microscope at × 400 magnification before the amoebae were collected. As shown in Fig. 1 morphological changes were observed after 8 h. Amoebae in NEFF saline solution as well as in Artelac® Splash presented the typical amoebae morphology attached to the culture flask with acanthopodia projecting and vacuolated cytoplasm, while amoebae in Optava Fusion™ were detached and rounded. In the culture bottle containing Oculotect®, only cellular debris was observed (Fig. 1).

### Viability tests

#### Trypan blue stain

Viability studied using Trypan Blue showed a decrease in the case of Optava Fusion™ and Artelac® Splash after the first 4 h. Nevertheless, after 8 h, recuperation of the amoebae was observed in both tears, amoebae concentration being similar to that of the NEFF control. In the case of Oculotect® *Acanthamoeba* trophozoites were not detected after 2 h of incubation.

#### Flow cytometry

**Fig. 2:**
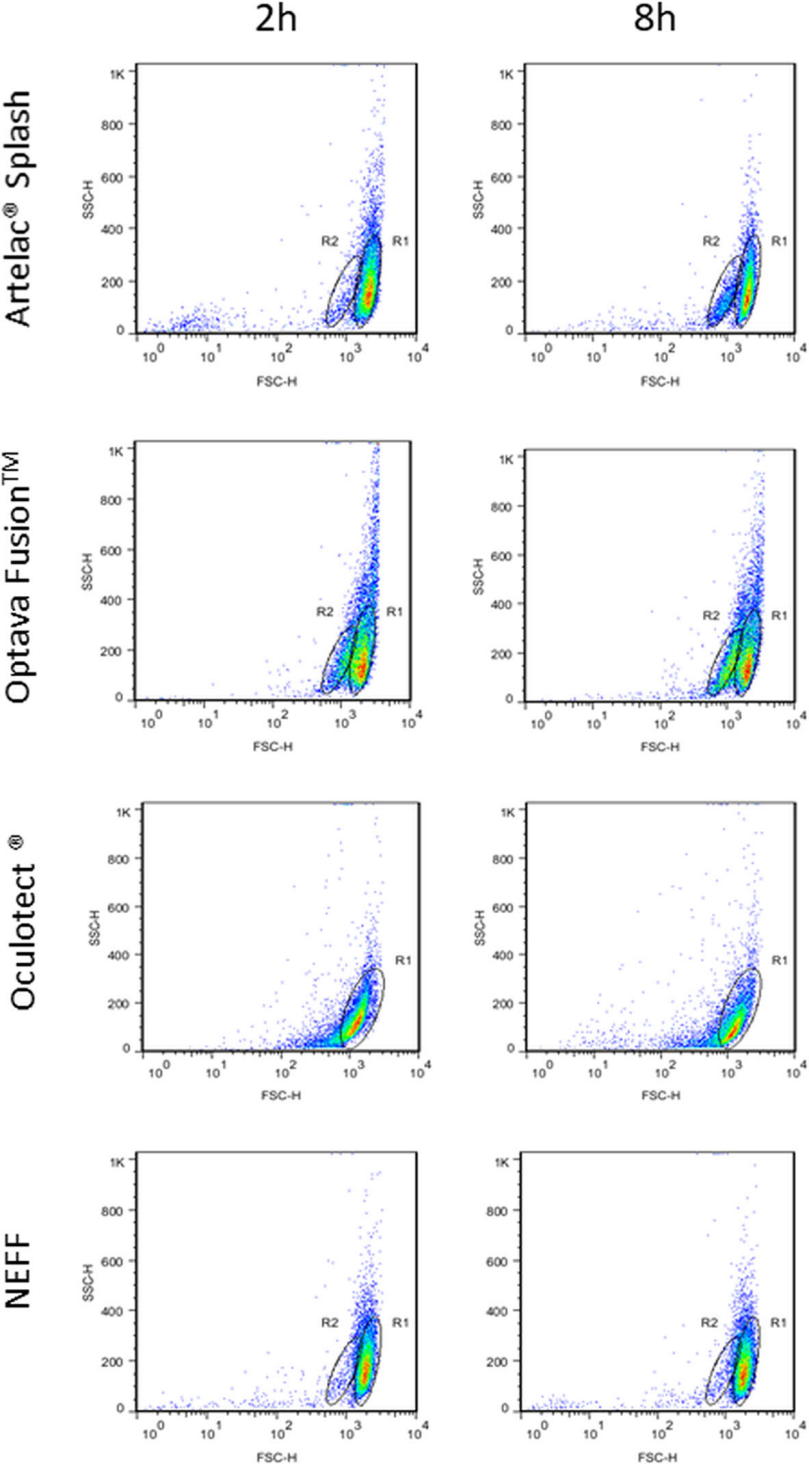
Histogram statistic of the CTC viability assay of Artelac® Splash, Optava Fusion™, Oculotect® and NEFF by flow cytometry at 2 h, 4 h, 6 h and 8 h incubation. R1: normal amoebae population; R2: altered smaller amoebae trophozoites

The flow cytometric analyses identified one population of parasites based on forward/side scatter values (R1) (Fig. 2). A second population of altered smaller trophozoites, R2, was observed after incubation with the artificial tears (Fig. 2), except for Oculotect® incubation where only an R1 population was observed for all the incubation times tested. After gating on the basis of forward/side scatter values, the quantification of the relative fluorescence intensity was determined for both populations of each sample. Based on the histogram representing the viability of standard trophozoites, an area (M1) was determined for the measurement of the percentage fluorescent amoebae in all samples. For the NEFF control, the percentage of viable amoebae decreased from 74.9% to 45.6% in 8 h (Table 1). After two hours’ treatment with Artelac® Splash and Optava Fusion™ a reduction of the viable population in R1 to 58.8 and 44.5% respectively, was observed. After 8 h, viability was 10.8 and 31.0%, respectively, which means a 4.22-fold and 1.47-fold increase with regard to NEFF. The percentage population of amoebae in R2 increased from 3.3 to 10.9% in 8 h for Artelac® Splash, and from 16.6 to 28.8% for Optava Fusion™. Treatment with Oculotect® for 2 h reduced the population of fluorescent amoebae to 4.9%, 15.35-times with regard to NEFF. The effect of Oculotect® was constant from the beginning and the percentage of viable amoebae remained similar at 8 h of incubation (4.1%) (Table 1).

## Discussion

In the present study, three artificial tear brands were selected according to the preservative agents (detergent or oxidative) or the lack of them to study their possible amoebicidal activity against trophozoites, as this is the initial state of the infection.

To study the viability of *Acanthamoeba* trophozoites after incubation with these tears, two different methods were used. The more traditional one, Trypan Blue stain, allows the differentiation of amoebae with the altered cell membrane and thus, identification of the non-viable ones. The second method used CTC stain to measure mitochondrial activity to determine the viability of the amoebae. This stain was developed for bacterial viability and then, adapted for *Acanthamoeba* in fluorometry by Kobayashi et al. [23]. In the present study, CTC stain for *Acanthamoeba* viability was used with flow cytometry for the first time.

One of the artificial tears tested using these two methods was Optava Fusion™. It contains Purite®, which is a soft oxidative preservative agent with demonstrated antimicrobial activity (bacteria, viruses and fungi), which dissociates on the eye surface in a photolytic reaction [24]. Contact of *Acanthamoeba* trophozoites with this artificial tear produced a slight decrease in the mitochondrial activity of amoebic cells (R1) during the incubation interval. However, a population of morphologically altered amoebae was observed which increased to 28.8% after eight hours (R2). If viability is considered as the sum of the two morphologies, results were similar to those obtained with Trypan Blue stain. In consequence, evaluation of both parameters, membrane integrity, and cell respiratory activity, seems to be crucial in the evaluation of the effectiveness of artificial tears and potential anti-amoebic compounds.

A further artificial tear evaluated was Artelac® Splash, whose formula does not contain any preservatives. Analysis of this artificial tear with Trypan Blue staining seemed to produce the same effect as the one observed in the presence of Optava Fusion™, demonstrating an apparent decrease in amoebae viability after two hours but a discrete increase for longer periods. However, analysis with flow cytometry and CTC staining revealed decreasing cell respiratory activity in the studied time intervals without morphological change (R1 and R2). Additionally, both Optava Fusion™ and Artelac® Splash contain sodium hyaluronate in their composition, a salt that has been proved to be a stimulator of proliferation of the corneal epithelium [25], so it could be also stimulating amoebic growth.

In the case of the artificial tear Oculotect®, the preservative agent used is BAK, a known antibiotic and amoebicidal compound [20]. This study demonstrated that BAK, in its commercially available formulation, maintains good amoebicidal activity despite the other components of the artificial tear and even when the tear is diluted 1:1. This amoebicidal effect was observed from the first hours of incubation and it maintained its effectiveness during all the time intervals evaluated with both viability techniques used. A decrease in the number of amoebic cells with Trypan Blue and low mitochondrial activity was observed using CTC.

In view of these results, we must highlight the importance of the data obtained with both methods used to investigate the viability of *Acanthamoeba* trophozoites. Trypan Blue staining and flow cytometry provide quite similar information for viability analysis, but they evaluate different characteristics, in such a way that the data they reveal are not exclusionary but complementary. As was observed with Artelac® Splash, some cells can diminish their mitochondrial activity gradually while maintaining the integrity of the plasmatic membrane, a characteristic that can be interpreted differently by these two methods if they are used separately. Our results demonstrate that conventional viability staining with Trypan Blue is a useful method. Nevertheless, flow cytometry analysis with CTC staining can be helpful in eliminating observer bias due to its automated feature and this prevents possible misevaluations caused by overexposure to Trypan Blue.

Finally, in this study, only Oculotect® was demonstrated to have an important and durable amoebicidal effect on *Acanthamoeba* trophozoites. However, we must be aware of the possible surface toxicity that BAK can produce on the cornea epithelium with frequent or extended use. This characteristic means that it is not advisable to use this artificial tear concomitantly with soft lenses. Hard contact lenses adsorb a small amount of this preservative and release a significant percentage of it during a regular washout procedure, approaching the upper safety limit, but soft contact lenses present a high adsorption of this substance. Such a property could allow the accumulation of BAK in the cornea epithelium, producing adverse effects [20]. Despite BAK’s toxicity, the promising results showed by Oculotect®, its use as a PrEP (pre-exposure prophylaxis) strategy before eye surgery should be considered. Nevertheless, studies in corneal models or even in patients are needed to establish the in vivo amoebicidal effect of this kind of artificial tear formulation.

## Conclusions

To our knowledge, the present study uses for the first time CTC stain analyzed by flow cytometry to establish *Acanthamoeba* viability demonstrating its usefulness and complementarity with the traditional stain, Trypan Blue. Artelac® Splash, with no preservatives, and Optava Fusion™, with Purite®, have not shown any amoebicidal activity. On the contrary, promising results shown by Ocultect®, with BAK, open up a new possibility of *Acanthamoeba* keratitis prophylaxis and treatment, although in vivo studies should be carried out.

## Abbreviations

AK: *Acanthamoeba* keratitis
BAK: Benzalkonium chloride
CL: contact lens
CTC: 5-cyano-2,3-ditolyl-tetrazolium chloride
DES: Dry eye syndrome
PYG: Protease peptone-yeast extract-glucose

## References

[1] del Buey MA, Cristobal JA, Casas P, Goni P, Clavel A, Minguez E, et al. Evaluation of in vitro efficacy of combined riboflavin and ultraviolet a for *Acanthamoeba* isolates. Am J Ophthalmol. 2012;153(3):399–404. 10.1016/j.ajo.2011.07.025 21992713

[2] Marciano-Cabral F, Cabral G (2003). *Acanthamoeba* spp. as agents of disease in humans. Clin Microbiol Rev.

[3] Yu HS, Choi KH, Kim HK, Kong HH, Chung DI (2001). Genetic analyses of *Acanthamoeba* isolates from contact lens storage cases of students in Seoul, Korea. Kor J Parasitol.

[4] Johnston SP, Sriram R, Qvarnstrom Y, Roy S, Verani J, Yoder J (2009). Resistance of *Acanthamoeba* cysts to disinfection in multiple contact lens solutions. J Clin Microbiol.

[5] Khan NA (2006). *Acanthamoeba*: biology and increasing importance in human health. FEMS Microbiol Rev.

[6] Lorenzo-Morales J, Khan NA, Walochnik J (2015). An update on *Acanthamoeba* keratitis: diagnosis, pathogenesis and treatment. Parasite.

[7] Page MA, Mathers WD (2013). *Acanthamoeba* keratitis: a 12-year experience covering a wide spectrum of presentations, diagnoses, and outcomes. J Ophthalmol.

[8] Panjwani N (2010). Pathogenesis Of *Acanthamoeba* keratitis. Ocul Surf..

[9] Lorenzo-Morales J, Martin-Navarro CM, Lopez-Arencibia A, Arnalich-Montiel F, Pinero JE, Valladares B (2013). *Acanthamoeba* keratitis: an emerging disease gathering importance worldwide?. Trends Parasitol.

[10] Visvesvara GS, Moura H, Schuster FL (2007). Pathogenic and Opportunistic free-living amoebae: *Acanthamoeba* spp., *Balamuthia mandrillaris*, *Naegleria fowleri*, and *Sappinia diploidea*. FEMS Immunol Med Microbiol.

[11] Kilvington S, Gray T, Dart J, Morlet N, Beeching JR, Frazer DG (2004). *Acanthamoeba* keratitis: the role of domestic tap water contamination in the United Kingdom. Invest Ophthalmol Vis Sci.

[12] McDonald M, Schachet JL, Lievens CW, Kern JR (2014). Systane(R) ultra lubricant eye drops for treatment of contact lens-related dryness. Eye Contact Lens.

[13] Fonn D (2007). Targeting contact lens induced dryness and discomfort: what properties will make lenses more comfortable. Optom Vis Sci.

[14] Zeev MS, Miller DD, Latkany R (2014). Diagnosis of dry eye disease and emerging technologies. Clin Ophthalmol.

[15] Narayanan S, Redfern RL, Miller WL, Nichols KK, McDermott AM (2013). Dry eye disease and microbial keratitis: is there a connection?. Ocul Surf.

[16] Kymionis GD, Bouzoukis DI, Diakonis VF, Siganos C (2008). Treatment of chronic dry eye: focus on cyclosporine. Clin Ophthalmol.

[17] Moshirfar M, Pierson K, Hanamaikai K, Santiago-Caban L, Muthappan V, Passi SF (2014). Artificial tears potpourri: a literature review. Clin Ophthalmol.

[18] Magnet A, Galvan AL, Fenoy S, Izquierdo F, Rueda C, Fernandez Vadillo C (2012). Molecular characterization of *Acanthamoeba* isolated in water treatment plants and comparison with clinical isolates. Parasitol Res.

[19] Caspers H, Page FC (1976). An illustrated key to freshwater and soil amoebae with notes on cultivation and ecology.

[20] Tu EY, Shoff ME, Gao W, Joslin CE (2013). Effect of low concentrations of benzalkonium chloride on acanthamoebal survival and its potential impact on empirical therapy of infectious keratitis. JAMA Ophthalmol.

[21] Beattie TK, Seal DV, Tomlinson A, McFadyen AK, Grimason AM (2003). Determination of amoebicidal activities of multipurpose contact lens solutions by using a most probable number enumeration technique. J Clin Microbiol.

[22] Magnet A, Pardinas C, García de Blas N, DS Gomez T, Saavada C, Carrillo E, et al. Anti-*Acanthamoeba* activity of different artificial tears used in the treatment of amoeba keratitis. In: VII European Congress of Protistology. Seville: Federation of European Protistological Societies; 2015. p. 158. http://www.semicrobiologia.org/protistologia/files/Book_of_Abstracts.pdf.

[23] Kobayashi T, Mito T, Watanabe N, Suzuki T, Shiraishi A, Ohashi Y (2012). Use of 5-cyano-2,3-ditolyl-tetrazolium chloride staining as an indicator of biocidal activity in a rapid assay for anti-*Acanthamoeba* agents. J Clin Microbiol.

[24] Charnock C (2006). Are multidose over-the-counter artificial tears adequately preserved?. Cornea.

[25] Aitken D, Hay J, Kinnear FB, Kirkness CM, Lee WR, Seal DV (1996). Amebic keratitis in a wearer of disposable contact lenses due to a mixed Vahlkampfia and Hartmannella infection. Ophthalmology.

